# Ultrasound Elasticity Imaging System with Chirp-Coded Excitation for Assessing Biomechanical Properties of Elasticity Phantom

**DOI:** 10.3390/ma8125458

**Published:** 2015-12-03

**Authors:** Guan-Chun Chun, Hsing-Jung Chiang, Kuan-Hung Lin, Chien-Ming Li, Pei-Jarn Chen, Tainsong Chen

**Affiliations:** 1 Department of Biomedical Engineering, National Cheng Kung University, Tainan City 70101, Taiwan; champ.chun@gmail.com (G.-C.C.); z7611641@gmail.com (H.-J.C.); qoo790525@gmail.com (K.-H.L.); 2 Division of Infectious Diseases, Department of Medicine of Chi Mei Medical Center, Tainan City 71004, Taiwan; 235813cmli@gmail.com; 3 Department of Electrical Engineering, Southern Taiwan University of Science and Technology, Tainan City 71005, Taiwan; cpj@stust.edu.tw

**Keywords:** Tukey window, chirp-coded pulse, cross-correlation, absolute difference

## Abstract

The biomechanical properties of soft tissues vary with pathological phenomenon. Ultrasound elasticity imaging is a noninvasive method used to analyze the local biomechanical properties of soft tissues in clinical diagnosis. However, the echo signal-to-noise ratio (eSNR) is diminished because of the attenuation of ultrasonic energy by soft tissues. Therefore, to improve the quality of elastography, the eSNR and depth of ultrasound penetration must be increased using chirp-coded excitation. Moreover, the low axial resolution of ultrasound images generated by a chirp-coded pulse must be increased using an appropriate compression filter. The main aim of this study is to develop an ultrasound elasticity imaging system with chirp-coded excitation using a Tukey window for assessing the biomechanical properties of soft tissues. In this study, we propose an ultrasound elasticity imaging system equipped with a 7.5-MHz single-element transducer and polymethylpentene compression plate to measure strains in soft tissues. Soft tissue strains were analyzed using cross correlation (CC) and absolution difference (AD) algorithms. The optimal parameters of CC and AD algorithms used for the ultrasound elasticity imaging system with chirp-coded excitation were determined by measuring the elastographic signal-to-noise ratio (SNRe) of a homogeneous phantom. Moreover, chirp-coded excitation and short pulse excitation were used to measure the elasticity properties of the phantom. The elastographic qualities of the tissue-mimicking phantom were assessed in terms of Young’s modulus and elastographic contrast-to-noise ratio (CNRe). The results show that the developed ultrasound elasticity imaging system with chirp-coded excitation modulated by a Tukey window can acquire accurate, high-quality elastography images.

## 1. Introduction

The standard medical practice of soft tissue palpation is based on the qualitative assessment of stiffness of tissues. Human tissue lesions are generally correlated with the variation in elastic properties of tissues. In many cases, despite the difference in stiffness between a lesion and normal tissue, the detection and evaluation of a pathological lesion via palpation is difficult owing to its small size or its location. In general, lesions may or may not exhibit sonographic contrast, which would enable them to be ultrasonically detectable. For instance, tumors of the prostate or breast may be significantly stiffer than the embedding tissue, and yet be invisible or barely visible in standard ultrasound examinations. Diffuse diseases such as cirrhosis of the liver are known to significantly increase the stiffness of liver tissue; however, the tissue may appear normal in conventional ultrasound examination [1].

Therefore, we can obtain new information related to the biomechanical properties of tissues for differentiating normal tissues from abnormal tissues by imaging tissue stiffness or a related parameter, such as strain under stress. In 1991, Ophir *et al.* [1] proposed a method to quantify strain information in biological tissues. A method capable of quantitatively imaging the stiffness of tissues with good resolution, sensitivity, and diminished speckle was presented in [2]. Ultrasound elastography can generate numerous types of images referred to as elastograms. Ultrasound elastography is typically used to evaluate a mass as benign or malignant for lesions. Soft and hard materials exhibit large and low strain values, respectively, and large and low strain values are typically displayed as bright and dark regions, respectively. Ultrasound elastography is being increasingly used for assessing the biomechanical properties of tissues and has been applied to numerous organs and pathologies including many cancers, such as scirrhous carcinoma of the breast, liver cancer, and prostatic carcinoma [3,4,5]. The different types of ultrasound elastography methods include compression ultrasound elastography, acoustic radiation force impulse (ARFI) technique, and shear wave elastography [5,6,7].

Compression ultrasound elastography is the most commonly used method in which a constant stress is applied to the studied tissue [5,7,8]. The technique uses manual compression or a controlled stepper motor to move a transducer to generate stress on the tissue and measures tissue deformation to estimate the elasticity of the tissue. Displacement is estimated by comparing echoes before and after compression by correlation methods [9]. The measured displacement, generated strain (ε), and stress can be used to compute the Young’s modulus, which is a more objective parameter of stiffness. This strain map is often referred to as an elastogram because the applied stress is unknown and only strain is displayed. Young’s modulus is the ratio of stress to strain within the elastic limit. Nightingale proposed the ARFI method in which acoustic radiation force is employed in place of transducer compression to generate force [7]. Acoustic radiation force is a unidirectional force applied to absorbing or reflecting targets in the propagation path of an acoustic wave. Attenuation is a frequency-dependent phenomenon and is primarily caused by absorption in soft tissues. The momentum transfer from the acoustic wave generates a force that causes displacement of the tissue [10,11]. Sarvazyan *et al.* [12] proposed a method, which can be considered to be a precursor of elastography techniques, based on ultrasonic radiation pressure by combining radiation pressure or acoustic radiation force and the shear waves generated. This technique is referred to as shear wave elasticity imaging (SWEI). Thereafter, Nightingale *et al.* validated the clinical feasibility of radiation force induced shear-wave imaging [13]. When acoustic radiation force is applied to a given spatial volume for a short duration, transient shear waves that propagate away from the initial region of excitation and perpendicular to the push pulse are generated. In tissues, shear waves travel at a velocity of approximately 1–10 m/s and can be easily tracked using ultrasound [10].

Ultrasound elastography is being increasingly used by clinicians to detect lesions or cancers in soft tissues. However, poor quality of elasticity imaging may degrade its value in clinical applications [14]. Imaging quality is determined by the visibility of small lesions and is limited by decorrelation noise [15]. Elastographic signal-to-noise ratio (SNRe), one of the major quality metrics of elasticity imaging, is affected by numerous factors such as echo signal-to-noise ratio (eSNR), applied strain, attenuation, penetration depth, and axial resolution. In an ultrasound system, the energy of the trigger signal affects eSNR and penetration depth, whereas the bandwidth of the signal influences axial resolution. A low eSNR reduces the uniformity between continuous echo frames acquired to estimate correlation-based displacement. Therefore, large displacement errors are generated and appear in strain images as a high-intensity noise; this is referred to as the decorrelation noise. In this situation, longer correlation window lengths reduce decorrelation noise for small tissue deformations at the cost of slightly-reduced axial resolution [16]. Therefore, the performance of elasticity imaging can be improved by increasing the energy of the trigger signal. In ultrasound imaging systems, the energy of the signal can be increased in two ways. First, when the peak power of the trigger signal is increased, commercial ultrasound systems increase peak power of trigger signal to the maximum value that is close to the mechanical index (MI) specification. Second, the average power of the trigger signal can be increased by lengthening the duration of the trigger. Therefore, a coded excitation signal can increase the average power of the trigger signal without affecting the power amplitude to realize a better eSNR.

Since the elastic properties of soft tissues are an important diagnostic indicator in clinics, palpation is one of the most commonly used diagnostic methods. However, palpation is limited to the inspection of superficial tissue, and the diagnosis is subjective with low sensitivity. The disadvantages of assessing tissue elasticity via palpation can be overcome by using ultrasound elastography. However, the eSNR is diminished because of the attenuation of ultrasonic energy by soft tissues, thus resulting in reduction of elastography qualities such as SNRe and elastographic contrast-to-noise ratio (CNRe). Chirp-coded pulse excitation has been proposed for improving the SNRe and CNRe of ultrasound elastography. However, the elongated chirp-coded pulses decrease the axial resolution of an ultrasound image. Therefore, pulse compression technique can be employed to improve the axial resolution of images. However, the effects of chirp-coded pulse modulated with different window functions and different strain analysis algorithms on elastography qualities are still not clearly known.

The aim of this study is to develop an ultrasound elasticity imaging system with chirp-coded excitation for assessing the biomechanical properties of tissue-mimicking phantom. Further, we analyzed the correlation between the qualities of elastography and a chirp-coded pulse modulated with different window functions and the effects of different strain analysis algorithms on the qualities of elastography.

## 2. System Architecture

**Figure 1:**
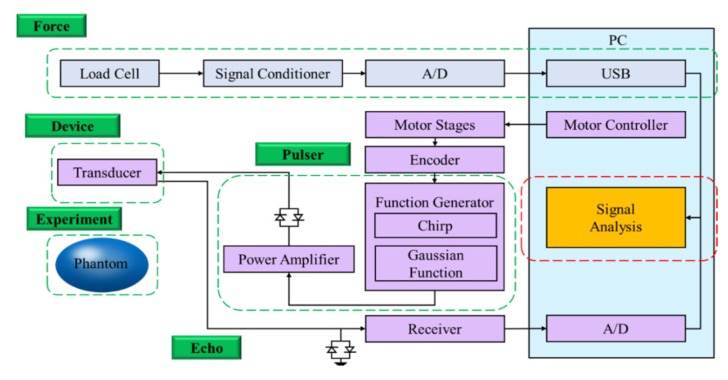
Block diagram of ultrasound elasticity imaging system.

In this study, an ultrasound elastography system was developed for quantitatively assessing the elasticity properties of tissues and phantoms. The schematic diagram of the proposed ultrasound elastography system with two types of excitation signals is shown in Figure 1. First, a 7.5-MHz single-element focused transducer (Model V320; Panametrics, Waltham, MA, USA) was excited by an ultrasonic pulser (Model 5058PR; Panametrics). Subsequently, a chirp waveform was generated using the MATLAB program and used as the trigger signal input for the arbitrary waveform generator (AWG; Model AFG3252; Tektronix Inc., Beaverton, OR, USA) connected to a radio-frequency power amplifier (Model 25A250A; Amplifier Research, Souderton, PA, USA). The echoes were then amplified by the receiver (Model 5073PR; Panametrics).

**Figure 2:**
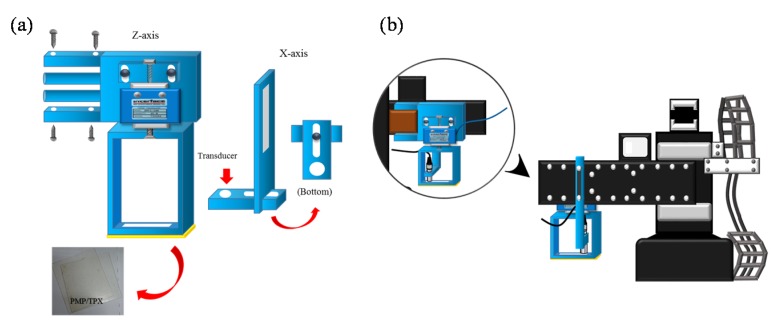
(**a**) Load cell and compression plate and (**b**) indentation system.

The received signals were digitized by an 8-bit analog-to-digital converter (Model PCI5152; National Instruments, Austin, TX, USA) at 2 GS/s sampling rate housed in a personal computer. The LabVIEW program was used to control the motor, acquire the data, and process the signals. Additionally, a three-axis step motor was used to move the ultrasonic probe and the indentation system containing the load cell (Model SMT S-Type; Interface Inc., Scottsdale, AZ, USA) and the compression plate (Polymethylpentene (PMP/TPX; Plastics Industry Development Center, Tainan City, Taiwan)), as shown in Figure 2a. The load cell was connected in series with a compression plate to record the corresponding force response. The indentation system was combined with a fixture and fixed on a *Z*-axis motor, and the ultrasonic probe was fixed on an *X*-axis motor, as shown in Figure 2b. 

## 3. Methodology Description

**Figure 3:**
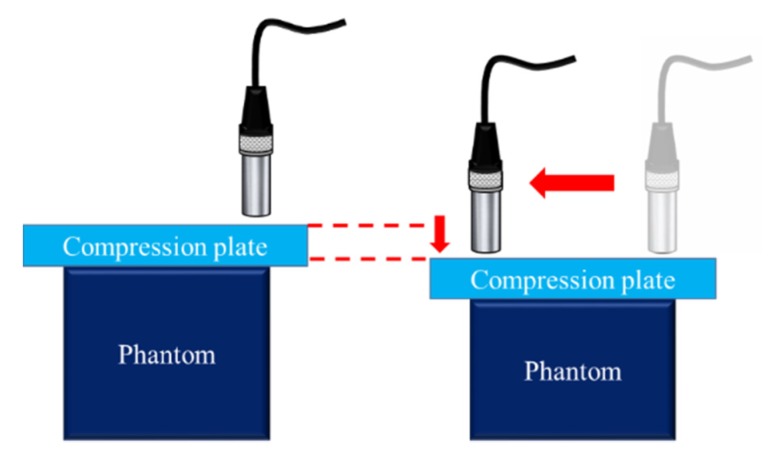
Measurement method.

The location of indentation system was adjusted by motor control interface which ensured the compression plate to press the object lightly. The position of the probe was controlled by step motor system for lateral scanning to construct two-dimensional B-mode image, as shown in Figure 3. A pre-compression radio frequency (RF)-signal was acquired during uniaxial loading and unloading of the phantom, respectively. Deformations of 1% of the phantom initial height were employed [2,17,18]. Finally, the software of MATLAB R2009a (The MathWorks, Natick, MA, USA) was used to process signal, and the elastic information of the object can be obtained. 

### 3.1. Chirp Pulse

A typical chirp signal is a linear frequency modulation waveform that means its instantaneous frequency is known as linear change with time [19], and linearly spans a frequency bandwidth *B* = *f_2_* − *f_1_*, where *f_1_* and *f_2_* are the starting and ending frequencies, respectively. If the chirp sweeps from *f_1_* to *f_2_* over a time, *T*, then chirp-code excitation is described by formula [14]:

(1) $$C\left(t\right)=w\left(t\right)\cos2\pi \left(f_{1}+\frac{B}{2T}t\right)t,\;\;\;\;\;0<t<T$$

where *w*(*t*) is a tapering window function. Signal duration is adjusted by compression ratio. When compression ratio increases, the signal duration is elongated. On the other hand, when compression ratio decreases, the signal duration is shortened. There are many window functions have be used for code pulse excitation. Such as Tukey, Hanning, Gaussian [14,15,20] *et al.*, in order to make the amplitude of the waveform is more prominent but the trigger signal maintains the same bandwidth. The window functions can be used as tapering or filtering window functions and many window functions have several parameters, it is nearly impossible to test all window functions to find the optimal chirp scheme for strain imaging.

**Figure 4:**
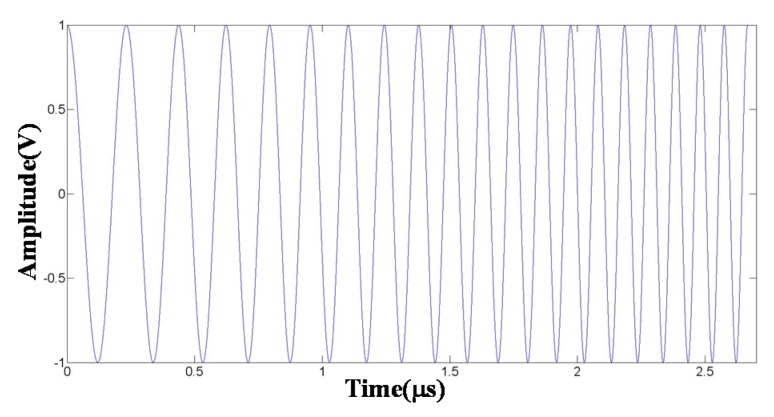
Chirp without window (*w*(*t*) = 1).

The chirp pulse was programed using the MATLAB program and input to the AWG that set the center frequency to be 7.5 MHz, as shown in Figure 4. In order to investigate the effect of window function and compression filter on strain imaging quality, two chirp schemes were designed, as shown in Table 1.

**Table 1:** Chirp schemes.

Name of Scheme	Window Function	Type of Compression Filter
Chirp 1	Tukey	Matched
Chirp 2	Gaussian	Matched

#### 3.1.1. Tukey Window

**Figure 5:**
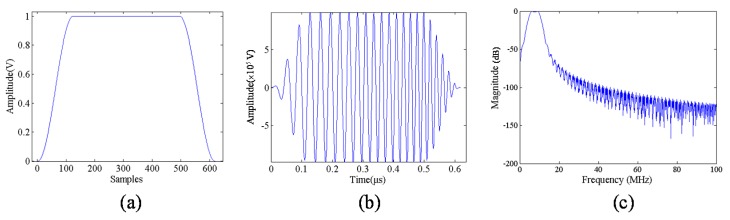
(**a**) Tukey window function; (**b**) chirp with Tukey window; and (**c**) frequency responses.

The Tukey window, often referred to as the cosine-tapered window, is shown in Figure 5. The window is defined as:

(2) $$w\left(t\right)\{\begin{array}{ll} 1.0, & 0\leq \left|t\right|\;\leq \;\beta \frac{T}{2}\\ \frac{1}{2}(1+\text{cos}\left(\pi \;\frac{\left|t\right|-\beta \left(T/2\right)}{2\left(1-\beta \right)\left(T/2\right)}\right)), & \beta \frac{T}{2}\;\leq \left|t\right|\leq \;\frac{T}{2} \end{array}$$

where *T* is the window length, and β ∈ (0, 1) such that β = 0 is a rectangular window and β = 1 is a Hanning window. Liu *et al.* reported that a chirp pulse excitation with a 40% Tukey window and matched compression filter exhibits better strain imaging performance [20].

#### 3.1.2. Gaussian Window

**Figure 6:**
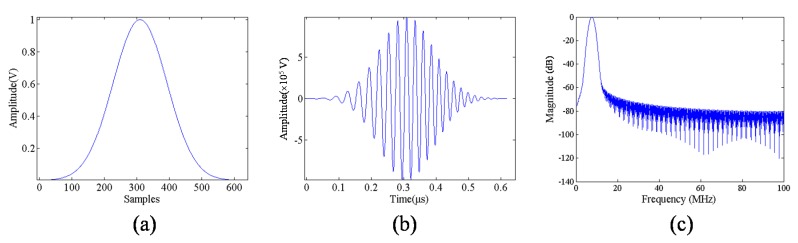
(**a**) Gaussian window function; (**b**) chirp with Gaussian window; and (**c**) frequency responses.

In mathematics, a Gaussian function, shown in Figure 6 and often simply referred to as a Gaussian, is a function of the form:

(3) $$w\left(t\right)=e^{\frac{1}{2}{\left(\alpha \frac{2t}{T-1}\right)}^{2}}$$

#### 3.1.3. Matched Filter

A matched filter is a common compression filter that eliminates phase difference to achieve compressed signals in response to extend pulse duration in the time domain. A matched filter can be defined as follows [21]:

(4) $$h\left(n\right)=s^{*}-n$$

**Figure 7:**
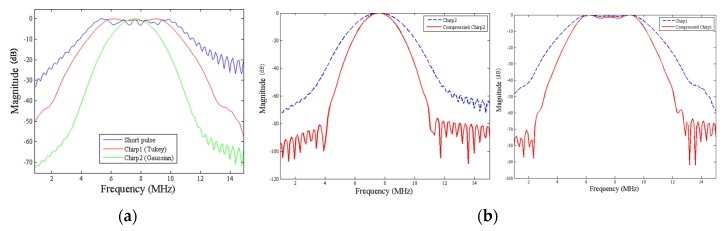
(**a**) Frequency responses before matched filter; and (**b**) frequency responses of Gaussian and Tukey after matched filter.

From Equation (3), the conjugate complex of *s(n)* shows that the phase of the matched filter is contrary to that of an echo signal, *, complex conjugate. Therefore, the phase difference can be eliminated by computing the convolution of *h(n)* and *s(n)*, thus focusing the echo signal energy to complete pulse compression , shown as Figure 7.

### 3.2. Tissue-Mimicking Phantom

Timothy *et al.* employed agar- and gelatin-based materials for elastography experimental validation [22]. The Young’s modulus values of these agar and gelatin dispersions are in the range of 5–135 kPa, a range representative of soft tissue, while the acoustic velocity and attenuation values are in the range of 1492–1575 m/s and 0.1–0.52 dB/(cm·MHz), respectively, again representative of soft tissue [23].

**Figure 8:**
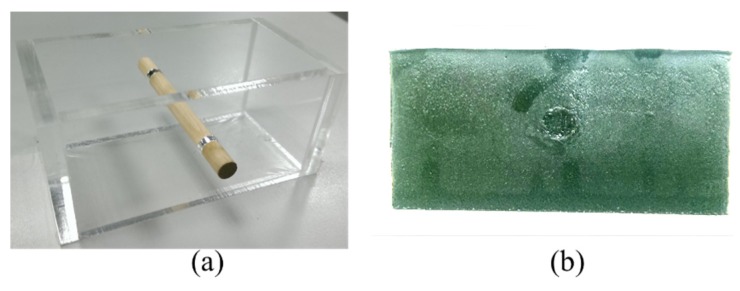
(**a**) A mold for constructing the block shaped phantom; (**b**) Phantom.

The block-shaped phantom used in this study was fabricated using a mold. A rod with 6 mm diameter was placed in a cubic mold with 40 mm height, 40 mm width, and 80 mm length, as shown in Figure 8. The phantom was fabricated using two different types of tissues to mimic normal and tumor tissues. In this study, the background material is an agar-gelatin mixture containing 7 wt % gelatin powder (SI-G2500, Uni-Onward Corp., New Taipei City, Taiwan) and 1 wt % agar powder (FLU-05093, Uni-Onward Corp.) dissolved in hot water. Tissue scattering was mimicked by graphite powder (AL-282863, Uni-Onward Corp.) of which 3 wt % was added to the gelatin/agar solution. A cylindrical tumor was fabricated using a mixture of 7 wt % gelatin powder, 2.5 wt % agar powder, and 3 wt % graphite powder. The agar-gelatin materials were produced according to the method described by Lopata *et al.* [22,24].

### 3.3. Signal Processor

**Figure 9:**
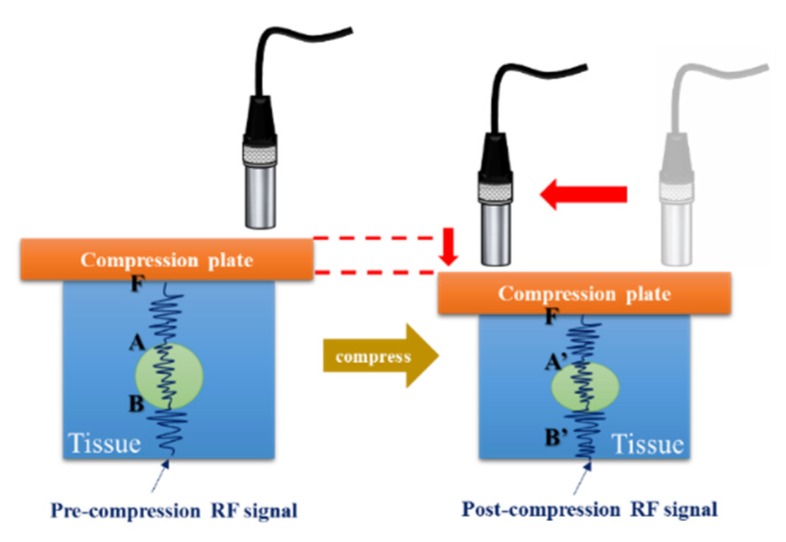
The schematic diagram of tissue before and after compression. RF: radio frequency.

**Figure 10:**
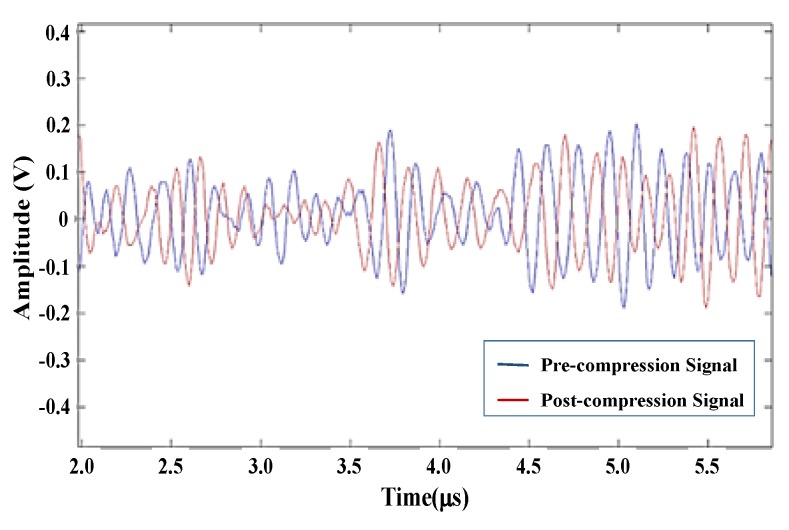
The signals of pre- and post-compression with time shift.

**Figure 11:**
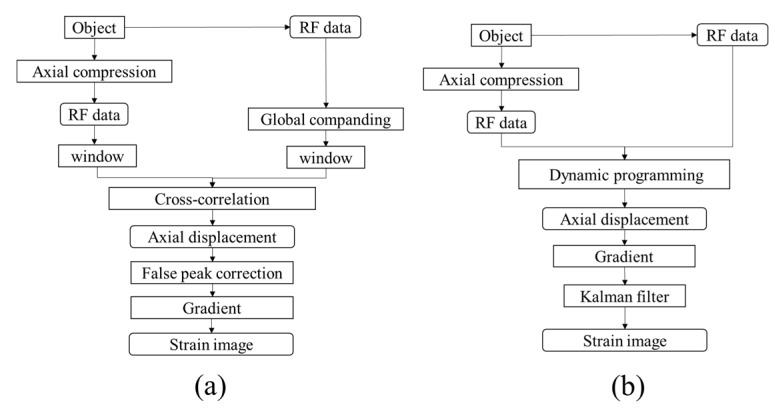
Flow chart of algorithms. (**a**) Cross correlation (CC); (**b**) Absolution difference (AD).

Objects with different elasticities exhibit different deformations under the same compression force. The ultrasound waves emitted from the transducer were used to measure the original tissue thickness and the variation in thickness of the soft tissue layer in the indented site on the basis of time-of-flight and sound speed. The average speed of sound in soft tissues of the human body is 1540 m/s. For instance, let us assume that the boundaries *A, B* are located on the path of the ultrasound waves emitted from the ultrasound transducer *F*, as shown in Figure 9. The distance between *A* and *B* is $\bar{AB}$ and $\bar{AB}=\bar{FA}-\bar{FA}$. Let us assume that the boundary *A* moves to *A′* and *B* moves to *B′* when pressure is exerted on the path of the ultrasound waves emitted from the transducer. The distance between *A′* and *B′* is $\bar{A\prime B\prime }$ and $\bar{AB}=\bar{AB}-\bar{A\prime B\prime }$. The waveform of echo signal varies when the object undergoes deformation under certain compression, as shown in Figure 10. Congruent A-lines were divided into temporal segments, and the corresponding time shifts of the segments were measured using cross correlation (CC) and absolution difference (AD) algorithms. Moreover, the Kalman filter was employed to estimate statistical noise such as white noise, as shown in Figure 11. 

#### 3.3.1. Cross Correlation Algorithm, CC

Before applying the cross correlation algorithm, the companding technique was used to correct the lateral displacement. Strain images are formed by analyzing pre-compression and post-compression echo fields *U* and C, respectively. Both the echo fields are Z×Y dimensional matrices of samples from continuous echo waveforms. For example, *U = (*$U_{z}$*,*$U_{y}$*)*; *z = 1… Z*; *y = 1…Y*, for *Y* waveforms each consisting of *Z* digitized echo values.

The sum of absolute differences (SAD) algorithm is implemented by selecting the corresponding data kernels from *U* and *C* of size L×M. For all *(i, j)* in a P×Q search region in *C* where *P* > *L* and *Q* > *M*, the SAD coefficients ($\in_{i,j}$) are computed as follows:

(5) $$\in_{i,j}=\overset{L}{\underset{l=1}{\sum }}\overset{M}{\underset{m=1}{\sum }}\left|C_{l,m}-U_{l+i,m+j}\right|$$

Let $\in_{i,j}$ = $\text{min}\left\{\in_{i,j}\right\}$ be the minimum SAD coefficient in the search region. The location of $\in_{i,j}$ indicates the position ($Z_{C},Y_{C}$) in *C* that corresponds to ($Z_{U},Y_{U}$) in *U*. 

Sixteen non-overlapping SAD kernels were equally distributed over *U* at points ($Z_{U},Y_{U}$). From the resultant displacement vectors, 16 corresponding points ($Z_{C},Y_{C}$) were estimated. Using linear regression analysis, the companding parameters $m_{y}$ and $m_{z}\;$and the shift parameters$\;b_{y}\;$and$\;b_{z}\;$were solved as follows:

(6) $$Z_{c}=m_{z}Z_{U}+b_{z},\;\;\;\;Y_{c}=m_{y}Y_{U}+b_{y}$$

We can use the CC algorithm to estimate the axial shift of the ultrasound echo signal. For continuous functions, the cross-correlation function is defined as follows [1]:

(7) $$R_{XY}\left(t\right)\overset{\mathrm{def}}{=}\int_{-\infty }^{\infty }X^{*}\left(\tau \right)Y\left(t+\tau \right)d\tau$$

where $R_{XY}\left(t\right)$ is the result of cross-correlation analysis, and X(τ) and Y(τ) are the precompression and post-compression radio frequency signals, respectively. When the functions match, the value of $R_{XY}\left(t\right)$ is maximized.

Cross-correlation is easy to implement. The only reason why cross-correlation is not used in real time applications is its computing load. In addition, cross-correlation can produce a “false peak” [22], that occurs when noise, finite window size, and signal decorrelation increase the amplitude of a secondary correlation peak above that of the primary correlation peak. False peaks are relatively large in magnitude and appear as discontinuities in the displacement vector. Thus, false peak correction is needful. Calculating the slope between two points, when slope exceeds a preset threshold, the previous point replaces the first point.

#### 3.3.2. Absolute Difference Algorithm, AD

Dynamic programming technique is used to estimate the displacement [13]. Let us consider two echo signals *g(i)* and *g′(i)* corresponding to the two A-lines acquired before and after compression. The difference between the two signals can be quantified using absolute differences as below:

(8) Δ(i,d)=|g(i)−g′(i+d)|

where $d_{\text{min}}\leq d\leq d_{\text{max}}$ is the displacement at the sample, and $d_{\text{min}}$ and $d_{\text{max}\;}$specify the allowed displacement. In ultrasound machines, the gains of radio frequency data can be altered to improve visualization. In order to reduce the effect of these variations on Δ, both pre-compression and post-compression ultrasound images are divided by the maximum value of one of the images. The smoothness of the displacements (*S*) is given as:

(9) $$S\left(d_{i},d_{i-1}\right)={\left(d_{i}-d_{i-1}\right)}^{k}$$

where $d_{i}$ is the displacement at sample *i*, and $d_{i-1}$ is the displacement at sample *i*−*1* of *g(i)*. The cost function *C* at the *i*th point and the associated displacement $d_{i}$ is defined as a recursive function as follows:

(10) $$C\left(i,d_{i}\right)=\underset{d_{i-1}}{\min}\left\{C\left(i-1,d_{i-1}\right)+wS\left(d_{i},d_{i-1}\right)\right\}+\Delta \left(i,d_{i}\right)$$

where *w* is a regularization weight which governs smoothness. 

The minimum of a scalar function of several variables must then be determined. The value of $d_{i-1}$ that minimizes is also memorized in a function for later use [19]:

(11) $$M\left(i,d_{i}\right)=\text{arg }\underset{d_{i-1}}{\min}\left\{C\left(i-1,d_{i-1}\right)+wS\left(d_{i},d_{i-1}\right)\right\}$$

nonlinear:

(12) {D(i)=arg mindi{C(i,di)}, i=mD(i)=M(i+1,D(i+1)), i=1…m−1

The displacements map of all the A-lines are independently calculated using the same procedure.

## 4. Experimental Results

### 4.1. Trigger Single Trial

**Figure 12:**
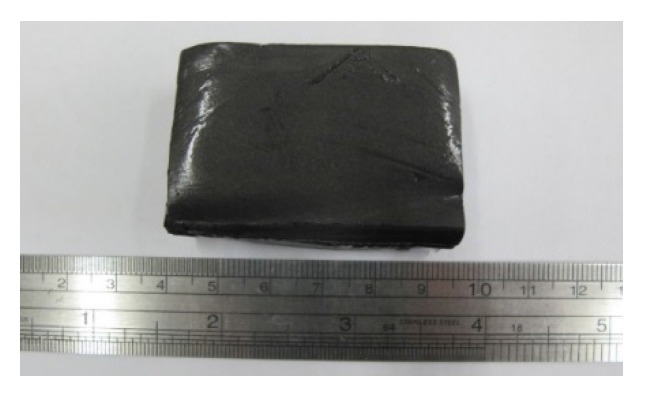
The photograph of a tissue-mimicking phantom.

In this thesis, a tissue-mimicking phantom, which is 50 mm long × 40 mm wide × 15 mm high is used. The tissue-mimicking phantom includes gelatin as a scattering factor to simulate the cell tissue of the human body. Figure 12 shows the photograph of the tissue-mimicking phantom. A 0.3-V chirp and short pulse with center frequency of 25 MHz as trigger signal is used to scan the tissue-mimicking phantom.

**Figure 13:**
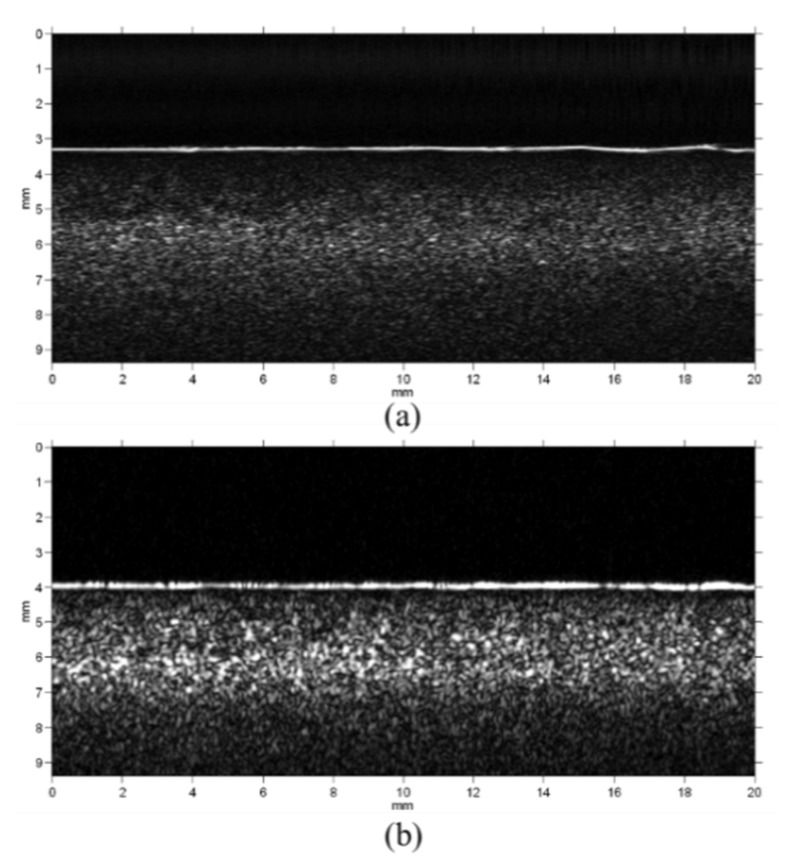
The images of tissue-mimicking phantom (**a**) short pulse and (**b**) chirp-coded excitation.

**Figure 14:**
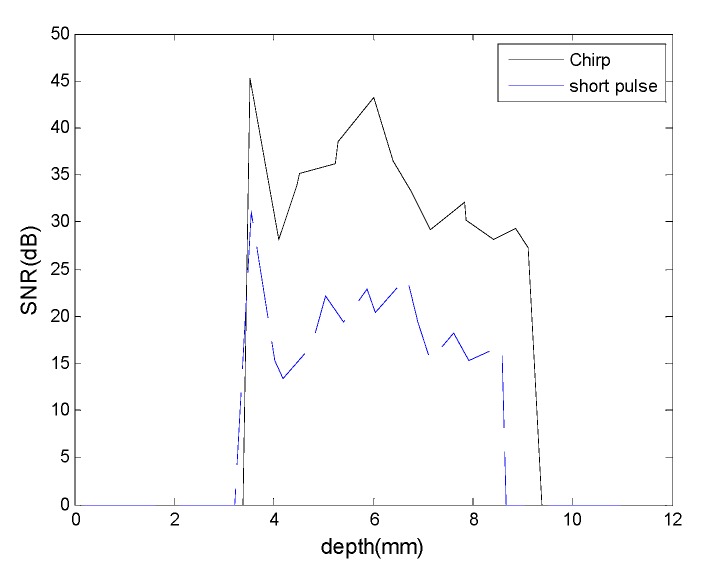
The signal-to-noise ratio as a function of depth.

Figure 13 shows the ultrasound images of the tissue-mimicking phantom by the short pulse imaging and chirp-coded excitation imaging. The image of the chirp-coded excitation is brighter than the image of the short pulse. For quantitative comparison, Figure 14 shows the signal-to-noise ratio as a function of depth from the middle A-line of both of the images. The average SNR of the chirp-coded excitation is about 40 dB and the average SNR of the short pulse is about 25 dB. There is a 15 dB SNR improvement by chirp-coded excitation.

**Figure 15:**
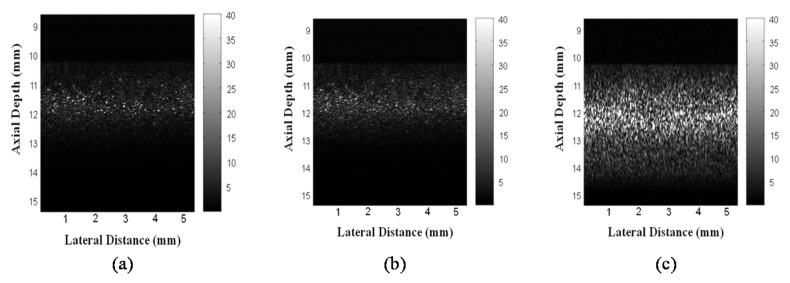
The images of the tissue-mimicking (**a**) unipolar pulse; (**b**) bipolar pulse; or (**c**) chirp-coded excitation.

The average power of the chirp signal is higher so the higher signal energy of the chirp signal is able to penetrate deeper into the tissue with the same tissue attenuation. Then pulse compression can recover the axial resolution and focus the energy of the signal to get stronger signal. Figure 15 shows the images of the tissue-mimicking phantom by unipolar pulse, bipolar pulse and chirp-coded excitation. There is a 1–2 mm penetration depth improvement by the chirp-coded excitation.

### 4.2. Strain-Stress Curve

Human soft tissue can be seen as a continuous material; therefore, it can be formulated as a sequential mechanics model. Hence, the stress-strain relations of human tissue can be modeled using the Hooke’s law. The relationship between stress and strain of a tendon is known to be in the longitudinal direction [25]. Moreover, the curve can be divided into four sections: toe area, linear elastic region, nonlinear elastic region, and inelastic region. In this study, we used scales and a three-axis step motor to record the force and compression distance under a pressure of 0.1 mm for each record reading of scale.

**Figure 16:**
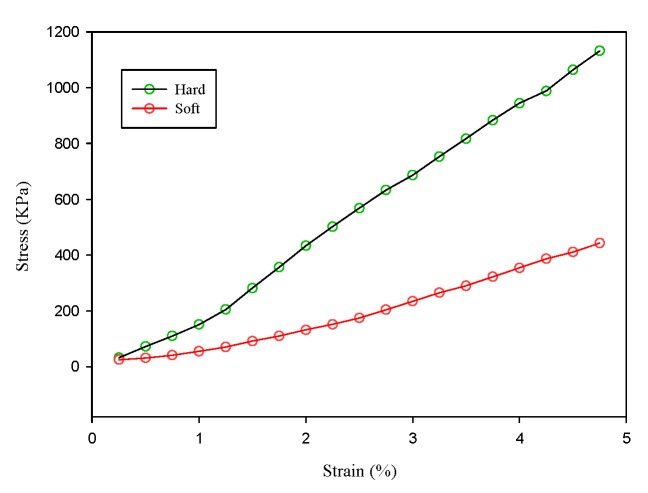
The strain-stress loading curves of two phantoms of different hardness.

The percentage strain values for each were plotted against the applied stress values. Figure 16 shows the experimental strain-stress loading curves of the phantoms. These curves are very similar to those mentioned above. Thereafter, we calculated the slope of the curve to obtain the Young’s modulus of the phantoms. The Young’s modulus values of the hard and soft phantoms are 26.86 and 13.27 kPa, respectively.

### 4.3. Optimal Parameters of Algorithm in Elastography

In order to accurately test the performance of chirp-coded pulse excitation for strain imaging, we employed two phantoms. One uniform phantom without any lesions was used for investigating the effect of pulse length, applied strain, and window length on strain imaging. Another lesion phantom having one lesion was used for testing the CNRe of the chirp pulse. Sections 4.3.1–4.3.3 describe the experimental method, parameters, and the results obtained using the uniform phantom, respectively. Sections 4.3.4–4.3.7 describe the experimental method, parameters, and the results obtained with the lesion phantom. A short pulse is a sine wave with a center frequency (*f*_0_) of 7.5 MHz. For a chirp pulse, the default center frequency is 7.5 MHz, and the starting and ending frequencies are 4 and 11 MHz, respectively.

#### 4.3.1. Effect of Pulse Length

**Figure 17:**
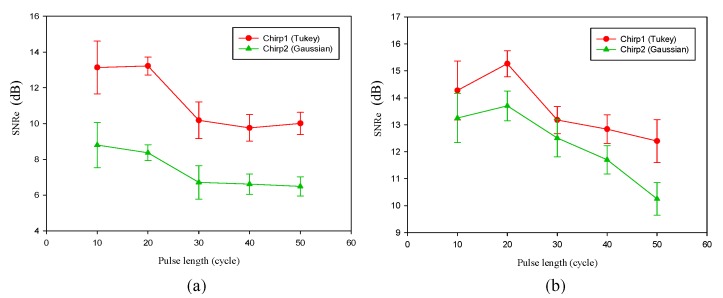
Elastographic signal-to-noise ratio (SNRe) for chirp pulse as a function of pulse length. Algorithm for (**a**) AD and (**b**) CC. The correlation window length is 0.77 mm.

A longer chirp pulse must be applied to achieve a larger eSNR gain. However, a longer pulse length does not necessarily ensure better SNRe. Figure 17 shows the optimal chirp pulse length for strain imaging. The longer the chirp pulse, the more distorted the received coded waveform owing to dynamic focusing [20,26]. When the pulse length is greater than 20 cycles, SNRe decreases with pulse length. Moreover, we can see that chirp 1 performs better than chirp 2. 

#### 4.3.2. Effect of Applied Strain

**Figure 18:**
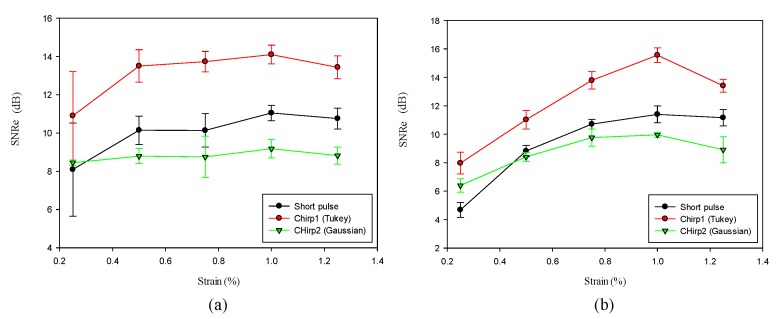
SNRe for all pulse as a function of applied strain. Algorithm for (**a**) AD and (**b**) CC. The correlation window length is 0.77 mm. The pulse length of chirp is 20 cycle.

Since the post-deformation echo signal is a function of displacements of tissue scatters, the performance of chirp pulse is also a function of applied strain. In order to determine the optimal applied strain for a chirp pulse, the applied strains were varied from 0.25% to 1.25%. Figure 18 shows that an optimal applied strain exists for chirp-coded strain imaging. The SNRe is the maximum when the strain is 1%, which is in accordance with previous investigation. In elastography, the applied compression is typically up to 1% of the total depth of the tissue [2] because a large applied strain will cause a large decorrelation noise of echo signal. However, for calculating strains, a large applied strain is less sensitive to displacement errors caused by system noise, finite window length, and signal decorrelation noise when compared with a small strain. Chirp 1 provides the greatest improvement over the short pulse. Conversely, the effect of chirp 2 is worse than that of the other two pulses.

#### 4.3.3. Effect of Correlation Window Length

**Figure 19:**
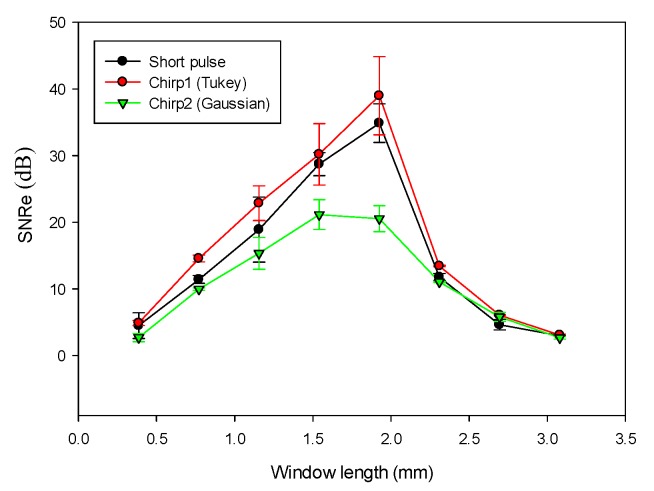
SNRe as a function of correlation window length.

For strain imaging, attainable axial strain resolution is largely determined by correlation window length [27]. A shorter window length will lead to better axial strain resolution than a long window length. In order to compare the performance of chirp and short pulses in terms of window length, we analyzed the effects of different window lengths on strain imaging for both short and chirp pulses. Figure 19 shows that the chirp 1 pulse requires a shorter window length to realize the same SNRe as that obtained with the short pulse. For example, to achieve a SNRe of 15 dB, the window length for short pulse is 1 mm, whereas it is only 0.77 mm for chirp 1 pulse. Therefore, the chirp pulse achieves a better axial strain resolution than the short pulse because the increased SNRe with the chirp pulse permits the use of a shorter window length. We selected a window length of 0.77 mm because the other window lengths are relatively unstable. Moreover, the smaller the window length, the higher is the axial resolution. However, chirp 2 performs poorly when compared with the other pulses. Although a long window length typically results in a higher SNRe, it also lowers the axial strain resolution. Therefore, a tradeoff exists between window length and axial strain resolution.

#### 4.3.4. CNRe

**Figure 20:**
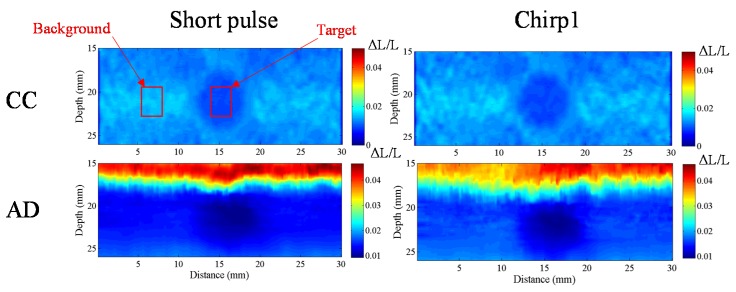
A strain image of short pulse and chirp pulse by CC and AD, respectively. The pulse length of chirp is 20 cycle. The correlation window length is 0.77 mm and applied strain is 1% are fixed.

**Figure 21:**
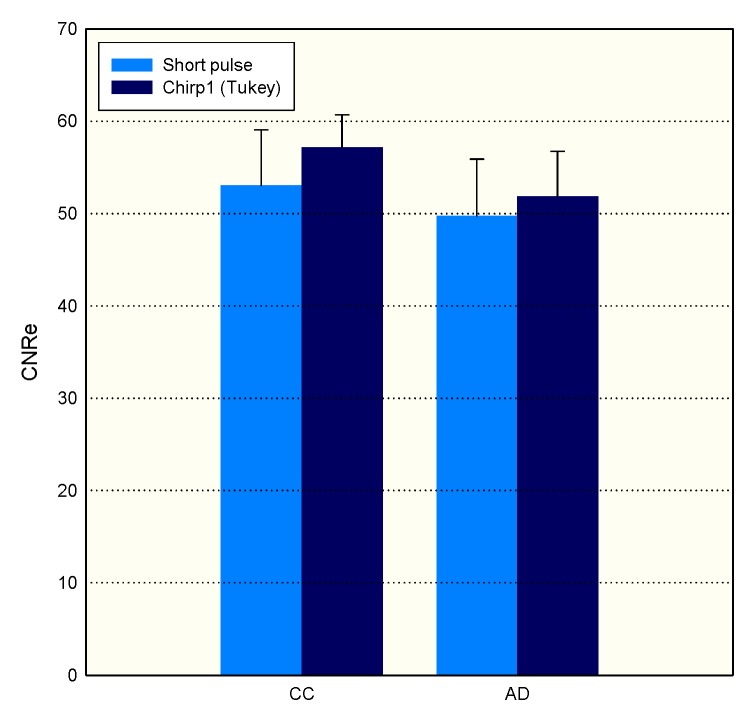
Elastographic contrast-to-noise ratio (CNRe) of phantom.

In order to investigate the CNRe of chirp and short pulses, we use the lesion phantom containing one lesion. Figure 20 shows the measured CNRe values for the lesion phantom in Figure 21. The CNRe values of the strain images obtained using AD algorithm are 49.76 ± 6.17 dB and 51.89 ± 4.87 dB for the short and chirp pulses, respectively. The CNRe values of the strain images obtained using the CC algorithm are 53.05 ± 6.03 dB and 57.18 ± 3.5 dB for the short and chirp pulses, respectively. Therefore, the chirp pulse achieves a slightly higher CNRe than the short pulse. Moreover, the short pulse is unstable when compared with the chirp pulse.

#### 4.3.5. Correlation Window Length

**Figure 22:**
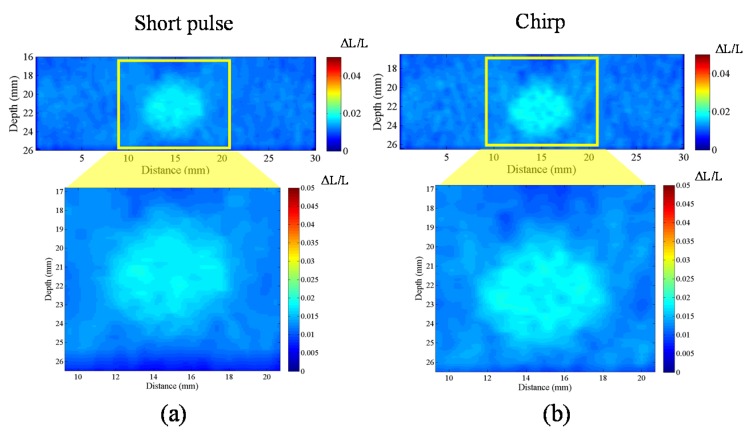
Strain images for two pulse. (**a**) The window length is 1 mm for short pulse, and the SNRe of the background and target is 15.99 and 19.69, respectively; and (**b**) the window length is 0.77 mm for chirp pulse, and the SNRe of the background and target is 16.03 and 20.12, respectively.

**Figure 23:**
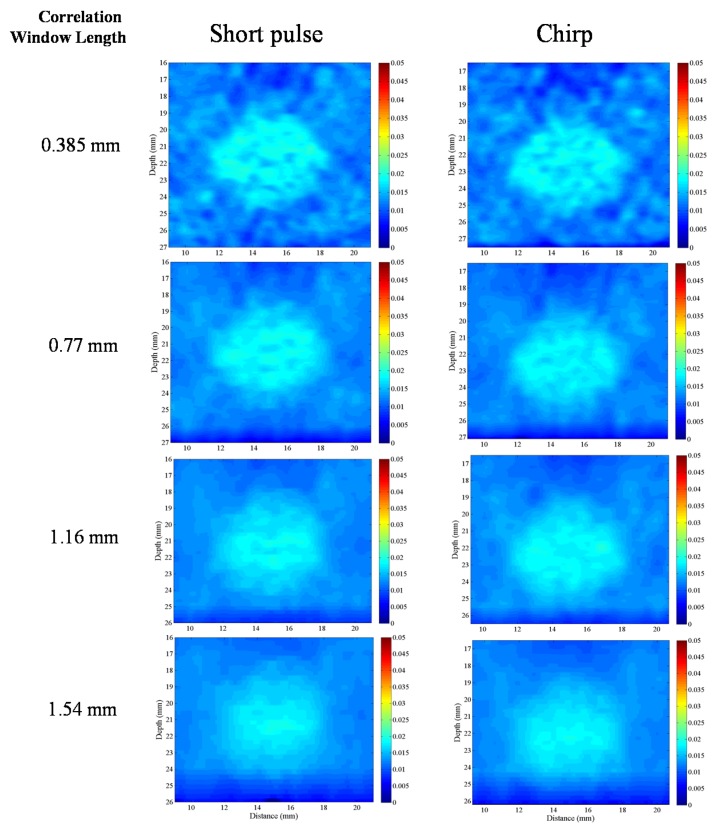
Strain imaging with different correlation window length. The correlation windows are 0.38, 0.77, 1.16 and 1.54 mm from top to bottom.

To validate the conclusion that a chirp pulse exhibits better axial strain resolution than a short pulse, we compare the axial strain resolutions of the chirp and short pulses on the lesion phantom. The SNRe values of the two backgrounds and targets in Figure 22 are nearly same; however, the window length for the short pulse in Figure 23a is 1 mm and that for the chirp pulse in Figure 22b is 0.77 mm. In order to compare the target in Figure 22, the target in Figure 22a is blurred more than the target in Figure 22b. Therefore, Figure 22 shows that the chirp pulse has a better axial strain resolution than the short pulse because the increased SNRe with the chirp pulse permits the use of a shorter pulse length.

Figure 23 shows a comparison of the strain images of the lesion phantom with 1% applied strain for short and chirp pulses for correlation window lengths of 0.385, 0.77, 1.16 and 1.54 mm. Using shorter correlation window, the strain image has higher resolution, it has more calculating operation time. The measured CNRe of the Figure 23 results are shown in Table 2. For the same correlation window length, the chirp pulses provide greater CNRe values than short pulses. Therefore, to achieve the same level of lesion detectability, the chirp pulse requires a shorter correlation window.

**Table 2:** Elastographic contrast-to-noise ratio (CNRe) measured for lesion phantom on Figure 23.

Windows Length (mm)	Short Pulse	Chirp Pulse
0.38	12.16	14.71
0.77	27.28	29.83
1.16	45.06	47.03
1.54	48.48	49.32

#### 4.3.6. Differential Strain

**Figure 24:**
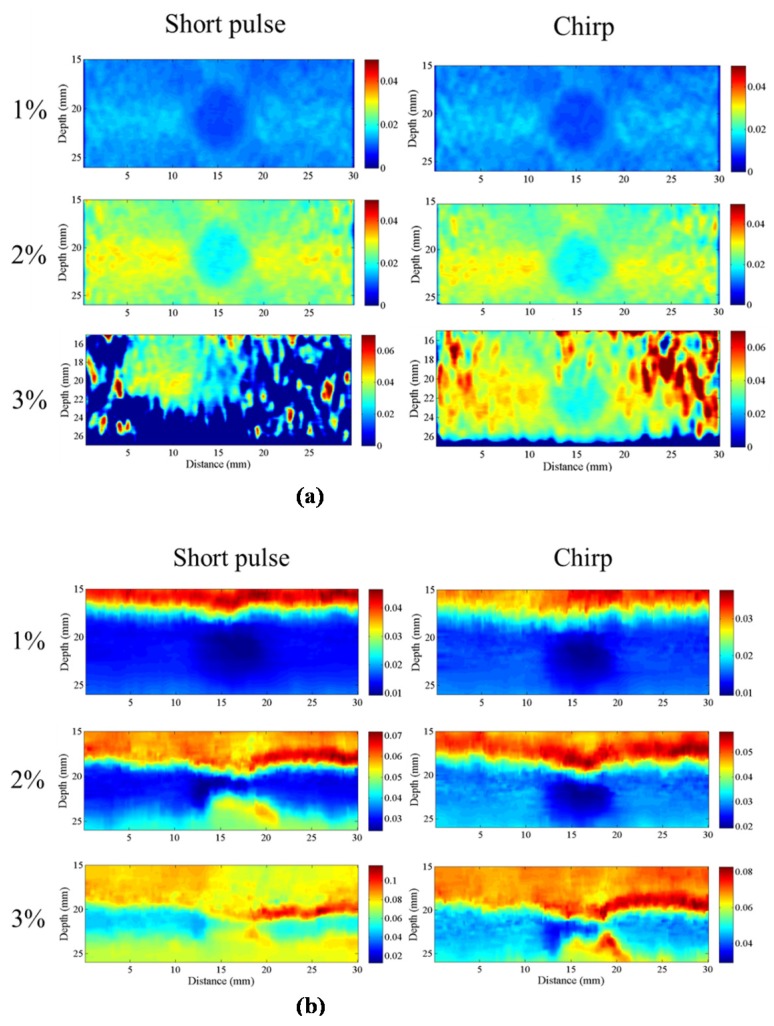
Strain images of a lesion phantom with 1%, 2% and 3% strain applied from top. (**a**) CC algorithm; and (**b**) AD algorithm.

In elastography, the applied strain is theoretically up to 1% of the total depth of the tissue [2]. When the strain is greater than 1%, decorrelation noise will appear in strain images. Figure 24 shows the strain images of the lesion phantom for applied strains of 1%, 2% and 3% using short and chirp pulses. The square boxes in Figure 24 indicate the regions from which the CNRe values shown in Table 3 are computed. As can be clearly seen, the chirp pulse successfully suppresses decorrelation strain noise.

**Table 3:** CNRe measured for lesion phantom on Figure 24.

Phantom	CC		AD	
Strain	Short	Chirp	Short	Chirp
1%	52.1	56.7	48.5	50.2
2%	40.8	42.3	13.2	16.1
3%	0.2	29.1	5.2	5.3

#### 4.3.7. Lateral Resolution

**Figure 25:**
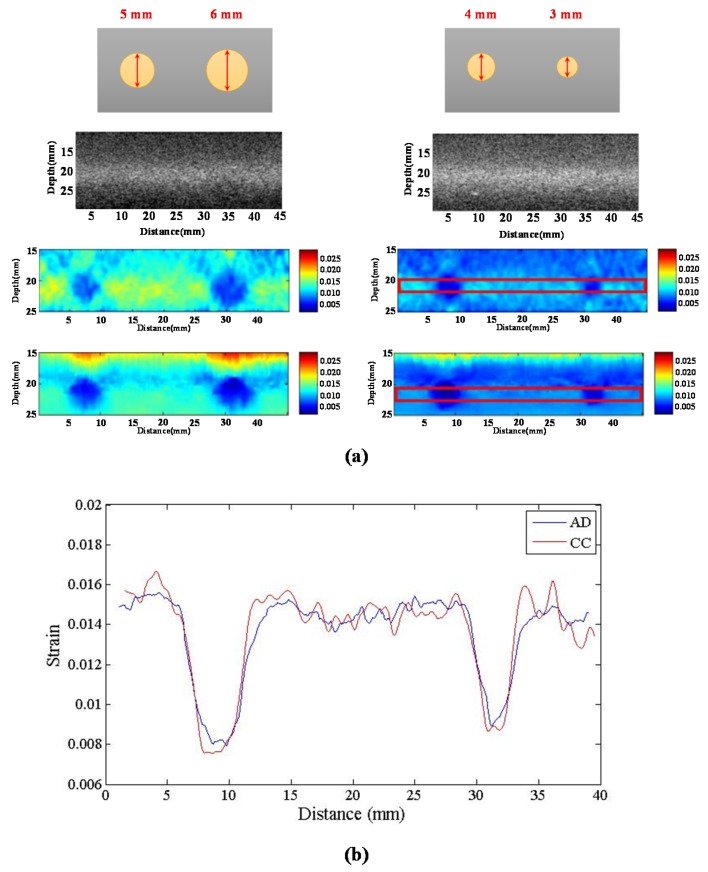
(**a**) Image of strain; and (**b**) curve of strain of strain image at 22 mm of depth.

In order to evaluate the accuracy of the system, we employed a hard cylinder embedded in the phantom at a depth of 20 mm at diameter of 6, 5, 4 and 3 mm as the scanning target. Figure 25a shows the strain images for the chirp pulse obtained using the two algorithms. We selected a few regions including the hard cylinder, whose locations and sizes are shown in Figure 25a, to compute the average strain profile of the elastogram. Figure 25b shows that the mean values of the estimates obtained using the CC algorithm are similar to those obtained using the AD algorithm. Further, the contrast of the strain image obtained using the AD algorithm is lower than that of the strain image obtained using the CC algorithm.

### 4.4. Young’s Modulus

According to above results, we used chirp pulse length, applied strain, and window length values of 20 cycles, 0.1%, and 0.77 mm, respectively, for all the measurements. The peak eSNR measured for our laboratory system is 30 dB for the lesion phantom. Considering the performances of chirp 1 and chirp 2 pulses, the chirp 1 scheme is applied to the ultrasonic strain imaging system for the phantom experiment. 

**Figure 26:**
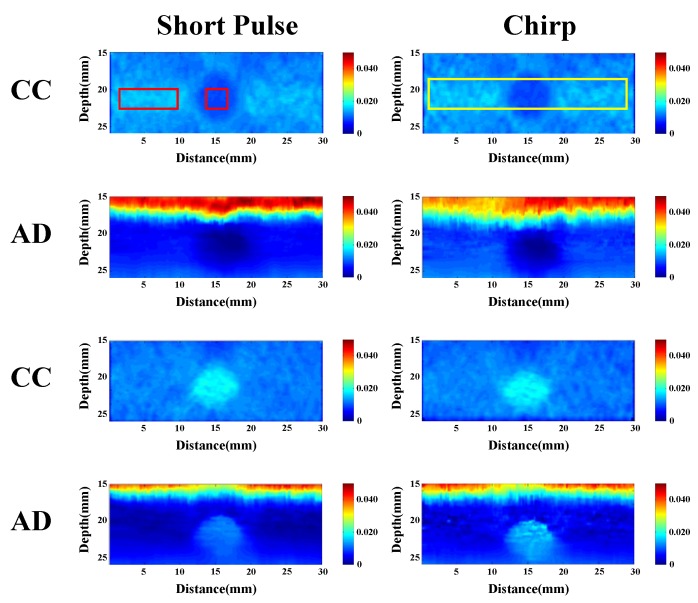
Strain image of two phantoms by CC and AD algorithm.

**Figure 27:**
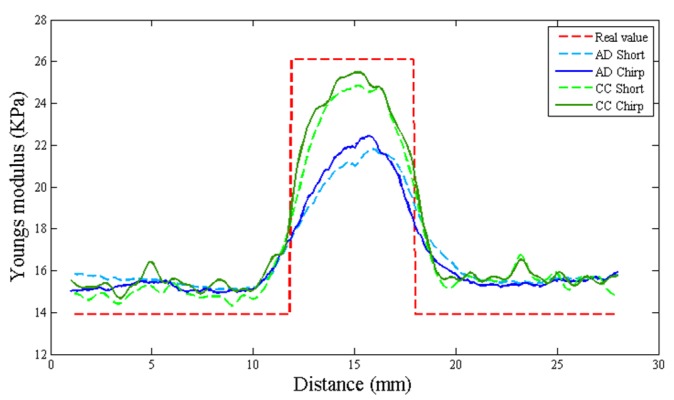
Young’s modulus of phantom including hard substance for two pulses by two algorithms.

Young’s modulus indicates the quality of stiffness. The phantoms were fabricated using different concentrations of agar. One phantom contained a cylinder softer than the background, and another phantom contained a cylinder harder than the background. Figure 26 shows the strain images of the two phantoms, and we can clearly see the different elasticities of the phantoms. In Figure 26, the red and yellow square boxes indicate the regions from which the Young’s modulus values shown in Table 4 and Figure 27 are computed, respectively.

**Table 4:** Young’s modulus of phantom.

Algorithm	Phantom	Short Pulse (kPa)	Chirp (kPa)
CC	Soft	14.50 ± 0.16	14.97 ± 0.66
	Hard	25.03 ± 0.77	25.52 ± 1.22
AD	Soft	14.66 ± 0.53	14.43 ± 0.08
	Hard	22.52 ± 0.70	22.72 ± 0.32

### 4.5. Discussion

The chirp pulse increases eSNR (30 dB) owing to its higher energy, and the increased eSNR improves the SNRe (15.7 dB). Therefore, the chirp pulse realizes a significantly higher SNRe than a short pulse (Figure 14 and Figure 15). The results of [17] show that some chirp schemes such as a chirp pulse excitation with 40% tapered Tukey window and matched compression filter performs better than short pulse scheme. Theoretically, the increased eSNR with chirp pulse should improve SNRe; however, the reason for the poor performance of the chirp 2 scheme when compared with that of the short pulse is unknown. Chirp pulse excitation with Gaussian window and matched compression filter is the most commonly used scheme for B mode imaging. However, Figure 14 and Figure 15 show that the common chirp scheme for B mode imaging is not the optimal scheme for elastograms. 

**Figure 28:**
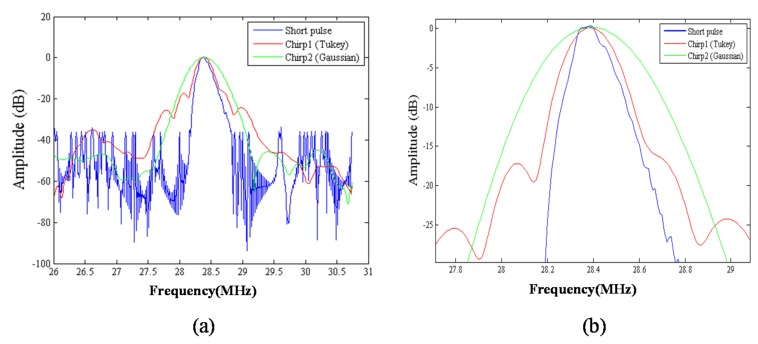
(**a**) Comparison of envelopes of short pulse, chirp 1 scheme and chirp 2 scheme with 20 cycles of pulse length; and (**b**) the detail in (**a**) from 27 to 29 MHz.

These results can be explained in terms of range side lobe level (RSLL) and main lobe width. The main lobe of the chirp scheme blurs pre-compressed and post-compressed echo signals; therefore, the broader the main lobe, the greater is the decorrelation noise caused by the main lobe. Figure 28 shows that the chirp 1 scheme is closer to the main lobe than the short pulse, and the chirp 1 scheme has a broader main lobe when compared with those of the other pulses. However, the chirp 1 scheme exhibits a higher RSLL than the short pulse. However, for the chirp 1 scheme, the normalized amplitudes of the side lobes except those close to the main lobe are below −20 dB. Therefore, the decorrelation noise due to the RSLL of chirp 1 scheme is small when compared with that due to the main lobe and can be neglected. On the other hand, the chirp 1 scheme exhibits greater eSNR than the short pulse and the chirp 2 scheme, as shown in Table 5. However, the main lobe width was not considered for evaluating the optimal chirp scheme for B mode imaging in [24]. Therefore, the performance of chirp pulse primarily depends on the main lobe width and energy. In strain imaging, an appropriate chirp scheme must be selected; otherwise, the chirp scheme may perform worse than the short pulse.

**Table 5:** The eSNR of three pulses.

Pulse	Short Pulse	Chirp 1 (Tukey)	Chirp 2 (Gaussian)
eSNR (dB)	20.22	33.09	30.36

A long chirp pulse penetrates deeper and achieves a higher eSNR gain than a short chirp pulse. However, the chirp pulse length is limited by dynamic receiving focusing, which distorts the received coded waveform prior to beam sum [26]. Figure 17 shows that an optimal pulse length exists for chirp-coded strain imaging. Therefore, we must select a short chirp pulse (approximately 20 cycles) for the excitation.

In the CC algorithm, correlation window length affects the attainable axial strain resolution. Since the chirp pulse leads to increased SNRe, it can use a shorter window length than short pulse to achieve the same SNRe, as shown in Figure 19. Therefore, the chirp pulse exhibits a better axial strain resolution than the short pulse. Moreover, Figure 18 and Figure 19 show that chirp pulse has better lesion detectability than the short pulse because of the higher CNRe, which is consistent with the results of [20]. Moreover, both the short and chirp pulses must have the same the lateral resolution, which is proportional to the beamwidth of the ultrasound system, because chirp coding and decoding procedures have no effect on the transducer aperture size [20]. Figure 13 and Figure 14 show that the optimal chirp pulse length is 20 cycles and the optimal strain is 1% of the total depth of the tissue for both the CC and AD algorithms. As can be seen in Figure 20, the CC algorithm performs better than the AD algorithm in elastograms because it can achieve a higher CNRe. Figure 24 shows that the optimal strain imaging can be achieved with a chirp pulse for excitation signal using the CC algorithm and over 1% strain. In order to compare the performances of the two algorithms, we plot the strain curve for the lesion phantom. Figure 25b shows that the strain curve corresponding to the AD algorithm is less obvious than that of the CC algorithm for the hard phantom. On the other hand, Figure 27 shows the elasticity distribution along the axial centerline of the phantom in Figure 26. The results of the phantom experiment indicate that the estimated and real values are significantly different, in accordance with the results in [28]. Makoto *et al.* reported that elasticity distribution can be reconstructed more quantitatively using a 3D model than with a two-dimensional (2D) model because these methods assume a 2D state, namely, a plane strain or plane stress state, which is not always satisfied in actual biological measurements [28]. The conventional method only considers axial and lateral displacement and the lack of positive displacement amount.

## 5. Conclusions

In this study, we proposed an ultrasound elastography system with chirp-coded excitation for evaluating the elasticity of a phantom (Young’s modulus of the background materials and cylindrical inclusion were 13.27 and 26.86 kPa, respectively) and investigating the strain performance of CC and AD algorithms. In order to minimize side lobes without degrading axial resolution, a mismatched compression filter was used to decode the echo signals.

Development of a chirp coded excitation ultrasound system is developed by using chirp signal as the ultrasound trigger signal in order to achieve better signal-to-noise ratio and deeper penetration depth. Table 6 shows that the echo signal of short pulse is less than the echo signal of chirp-coded excitation. According to experiment results and discussion, there are a 15 dB SNR improvement and a 1–2 mm penetration depth improvement by chirp-coded excitation, pulse compression, the appropriate receiver and the handmade expander of noise reduction and SNR enhancement.

**Table 6:** The relationship between the pulse type and echo peak amplitude.

Pulse Type	Peak Frequency (MHz)	Echo Peak Amplitude (V)
Unipolar Pulse	25	0.96
Bipolar Pulse	25	1.04
Coded excitation (Chirp)	25	2.08

We compared the performance of different chirp schemes with the conventional short pulse. The chirp with Gaussian window, which is common and suitable for B-mode imaging, was found to exhibit poor performance of SNRe than both the short pulse and the chirp with Tukey window because the width of the main lobe was not been considered for evaluating the optimal chirp scheme for B-mode imaging. Thereafter, the effects of different factors such as chirp pulse length, applied strain, and correlation window length on strain imaging were investigated. Optimal parameters of the three pulses were the same for both the CC and AD algorithms. 

The homogenous phantom experimental results show that the SNRe of elastography measured using a short pulse is 11 dB. The SNRe values measured using a 20-cycle chirp-coded ultrasound system modulated using CC and AD algorithms were 15 and 13 dB, respectively. The CNRe of the image obtained using the chirp-coded pulse can be improved by 4.1 dB when compared with that obtained using the short pulse. The results validate that a chirp with Tukey window has better lesion detectability than a short pulse. Additionally, the Young’s modulus values of the cylindrical inclusion analyzed using the CC and AD algorithms were 25.52 and 22.72 kPa, respectively. These results show that the ultrasound elasticity imaging system with chirp-coded excitation modulated by a Tukey window can acquire highly accurate and high quality elastography images. In the future, the ultrasound elasticity imaging system will be used on human subjects to validate the feasibility of collecting *in vivo* data [29,30,31].

## References

[1] Ophir J., Alam S.K., Garra B.S., Kallel F., Konofagou E.E., Krouskop T., Merritt C.R.B., Righetti R., Souchon R., Srinivasan S. (2002). Elastography: Imaging the elastic properties of soft tissues with ultrasound. J. Med. Ultrason..

[2] Ophir J., Cespedes I., Ponnekanti H., Yazdi Y., Li X. (1991). Elastography: A quantitative method for imaging the elasticity of biological tissues. Ultrason. Imaging.

[3] Zhai L., Madden J., Foo W.C., Palmeri M.L., Mouraviev V., Polascik T.J., Nightingale K.R. (2010). Acoustic radiation force impulse imaging of human prostates *ex vivo*. Ultrasound Med. Biol..

[4] Gallotti A., D’onofrio M., Romanini L., Cantisani V., Mucelli R.P. (2012). Acoustic Radiation Force Impulse (ARFI) ultrasound imaging of solid focal liver lesions. Eur. J. Radiol..

[5] Balleyguier C., Canale S., Hassen W.B., Vielh P., Bayou E.H., Mathieu M.C., Uzan C., Bourgier C., Dromain C. (2013). Breast elasticity: Principles, technique, results: An update and overview of commercially available software. Eur. J. Radiol..

[6] Destounis S., Gruttadauria J.L. (2013). Elasticity imaging 101. J. Radiol. Nurs..

[7] Gennisson J.L., Deffieux T., Fink M., Tanter M. (2013). Ultrasound elastography: Principles and techniques. Diagn. Interv. Imaging.

[8] Varghese T. (2009). Quasi-static ultrasound elastography. Ultrasound Clin..

[9] Gao L., Parker K.J., Lerner R.M., Levinson S.F. (1996). Imaging of the elastic properties of tissue—A review. Ultrasound Med. Biol..

[10] Benson J., Fan L. (2012). Tissue Strain Analytics—A Complete Ultrasound Solution for Elastography. http://www.google.com/url?sa=t&rct=j&q=&esrc=s&source=web&cd=1&ved=0ahUKEwjRqMarqKvJAhUKJ5QKHZ8CDd0QFggjMAA&url=http%3A%2F%2Fsonoworld.com%2FCommon%2FDownloadFile.aspx%3FModuleDocumentsId%3D66&usg=AFQjCNFvP5Ymuj2jd1BYncxUJoXKBSF9Xg&bvm=bv.108194040,d.dGo&cad=rja.

[11] Nightingale K.R., Palmeri M.L., Nightingale R.W., Trahey G.E. (2001). On the feasibility of remote palpation using acoustic radiation force. J. Acoust. Soc. Am..

[12] Sarvazyan A.P., Rudenko O.V., Swanson S.D., Fowlkes J.B., Emelianov S.Y. (1998). Shear wave elasticity imaging: A new ultrasonic technology of medical diagnostics. Ultrasound Med. Biol..

[13] Nightingale K., McAleavey S., Trahey G. (2003). Shear-wave generation using acoustic radiation force: *In vivo* and *ex vivo* results. Ultrasound Med. Biol..

[14] Peng H., Liu D.C. Chirp-coded pulse excitation for ultrasound elasticity imaging. Proceedings of the 2010 4th International Conference on Bioinformatics and Biomedical Engineering (iCBBE).

[15] Liu J., Insana M.F. (2005). Coded pulse excitation for ultrasonic strain imaging. IEEE Trans. Ultrason. Ferroelectr. Freq. Control.

[16] Chaturvedi P., Insana M.F., Hall T.J. (1998). 2-D companding for noise reduction in strain imaging. IEEE Trans. Ultrason. Ferroelectr. Freq. Control.

[17] Cespedes I., Ophir J., Ponnekanti H., Maklad N. (1993). Elastography: Elasticity imaging using ultrasound with application to muscle and breast *in vivo*. Ultrason. Imaging.

[18] Fung Y.C. (1993). Biomechanics: Mechanical Properties of Living Tissues.

[19] Qiu W., Yu Y., Tsang F.K., Zheng H., Sun L. (2013). A novel modulated excitation imaging system for microultrasound. IEEE Trans. Biomed. Eng..

[20] Peng H., Liu D.C. (2013). Enhanced ultrasound strain imaging using chirp-coded pulse excitation. Biomed. Signal Process. Control.

[21] Turin G. (1960). An introduction to matched filters. IRE Trans. Inf. Theory.

[22] Zahiri-Azar R., Salcudean S.E. (2006). Motion estimation in ultrasound images using time domain cross correlation with prior estimates. IEEE Trans. Biomed. Eng..

[23] Chaturvedi P., Insana M.F., Hall T.J. (1998). Testing the limitations of 2-D companding for strain imaging using phantoms. IEEE Trans. Ultrason. Ferroelectr. Freq. Control.

[24] Varghese T., Ophir J. (1997). Enhancement of echo-signal correlation in elastography using temporal stretching. IEEE Trans. Ultrason. Ferroelectr. Freq. Control.

[25] Price J.M., Patitucci P., Fung Y.C. (1981). Biomechanics. Mechanical Properties of Living Tissues.

[26] O’Donnell M. (1992). Coded excitation system for improving the penetration of real-time phased-array imaging systems. IEEE Trans. Ultrason. Ferroelectr. Freq. Control.

[27] Liu J., Abbey C.K., Insana M.F. (2004). Linear approach to axial resolution in elasticity imaging. IEEE Trans. Ultrason. Ferroelectr. Freq. Control.

[28] Yamakawa M., Shiina T. (1999). Tissue elasticity reconstruction based on 3-dimensional finite-element model. Jpn. J. Appl. Phys..

[29] Hu C.H., Liu R., Zhou Q., Yen J., Shung K.K. (2006). Coded excitation using biphase-coded pulse with mismatched filters for high-frequency ultrasound imaging. Ultrasonics.

[30] Santos S.D., Domenjoud M., Prevorovsky Z. (2010). Ultrasonic imaging of human tooth using chirp-coded nonlinear time reversal acoustics. Phys. Procedia.

[31] Pedersen M.H., Misaridis T.X., Jensen J.A. (2003). Clinical evaluation of chirp-coded excitation in medical ultrasound. Ultrasound Med. Biol..

